# Approaches Toward Targeting Matrix Metalloproteases for Prognosis and Therapies in Gynecological Cancer: MicroRNAs as a Molecular Driver

**DOI:** 10.3389/fonc.2021.720622

**Published:** 2022-01-25

**Authors:** Anuradha Pandit, Yasmin Begum, Priyanka Saha, Amit Kumar Srivastava, Snehasikta Swarnakar

**Affiliations:** ^1^ Infectious Diseases & Immunology Division, CSIR-Indian Institute of Chemical Biology, Kolkata, India; ^2^ Cancer Biology & Inflammatory Disorder Division, CSIR-Indian Institute of Chemical Biology, Kolkata, India

**Keywords:** microRNA, gynecological cancer, matrix metalloprotease (MMP), EMT, metastasis

## Abstract

Gene expression can be regulated by small non-coding RNA molecules like microRNAs (miRNAs) which act as cellular mediators necessary for growth, differentiation, proliferation, apoptosis, and metabolism. miRNA deregulation is often observed in many human malignancies, acting both as tumor-promoting and suppressing, and their abnormal expression is linked to unrestrained cellular proliferation, metastasis, and perturbation in DNA damage as well as cell cycle. Matrix Metalloproteases (MMPs) have crucial roles in both growth, and tissue remodeling in normal conditions, as well as in promoting cancer development and metastasis. Herein, we outline an integrated interactive study involving various MMPs and miRNAs and also feature a way in which these communications impact malignant growth, movement, and metastasis. The present review emphasizes on important miRNAs that might impact gynecological cancer progression directly or indirectly *via* regulating MMPs. Additionally, we address the likely use of miRNA-mediated MMP regulation and their downstream signaling pathways towards the development of a potential treatment of gynecological cancers.

## Background

Gynecological malignancies, like cervical, ovarian, and endometrial cancers, account significantly for most of the global cancer load, where cervical cancer (CC) accounts to be the fourth most prevalent malignancy among women, along with ovarian cancer (OC) comprising 4.4% of the entire cancer-related mortality among women (1). In 2018, endometrial cancer (EC) was reported to have caused 382,069 cases and 89,929 deaths globally (1). The percentage of women over 65 diagnosed with cancer is projected to increase dramatically over the next decade (2). As a result, there is already a significant unmet therapeutic need in the field for successful treatments of gynecological malignancies.

Gynecological cancers have a high mortality rate due to the diagnosis at late stages in addition to multi-drug resistance, impaired apoptotic pathway, inhibition of the immune system, and aberrant MMP production (3, 4). Extracellular matrix (ECM) remodeling is crucial for maintaining extracellular microenvironment homeostasis and tissue turnover. Tumor cells must be able to disrupt the surrounding ECM to proliferate, invade, and metastasize. Uncontrolled tumor proliferation, tissue remodeling, inflammation, cellular invasion, and metastasis are all consequences of abnormal ECM proteolysis. Matrix metalloproteases (MMPs) are enzymes capable of degrading multiple ECM components, leading to wound healing, tissue repair, embryonic development (5). Rampant MMP expression has been associated with tumor aggressiveness, metastasis, and vascularization and is correlated with late diagnosis in various malignancies such as lung, prostate, colon, breast, and pancreatic cancers (6–10). MMP expression is closely monitored by many regulatory mechanisms, which include zymogen activation, compartmentalization, endogenously produced tissue inhibitors of metalloproteases (TIMPs), and miRNAs.

miRNAs are endogenously produced non-coding RNA elements responsible for gene silencing by degrading target mRNA. They are frequently altered during tumorigenesis and their ability to regulate various genes has made them an attractive candidate for cancer therapeutics (11). Dysregulation of both MMP and miRNA levels is a pronounced feature of gynecological cancers (12–14). The involvement of miRNAs to regulate the expression of the MMP gene has recently received a lot of attention. MMP regulation by various miRNAs may affect cancer progression. Moreover, the functional relevance of miRNA-mediated MMP regulation in malignancies might be explored further by examining the post-transcriptional regulation system controlling MMP gene expression. The current study focuses on the mechanisms controlling MMP expression by miRNAs in gynecological cancers and also aims to come up with a strategy to assist miRNAs targeting MMPs for diagnosis and therapeutic intervention.

## miRNA Biogenesis

Numerous small RNAs have been evolved to negatively regulate undesired genetic elements and transcripts (15). miRNAs are the most dominating group of small RNAs having a length of ~22 nucleotides and are generated by RNase III proteins namely Dicer and Drosha (16). miRNA functions as a guide by targeting specific mRNAs at its 3’untranslated region (3’UTR) region usually by base-pairing thereby inducing RNA silencing (17) and AGO proteins act as the effector proteins recruiting factors that induce mRNA deadenylation, translational repression, and mRNA degradation (18).

Because each miRNA affects a vast number of mRNAs, the miRNA biogenesis pathway has a pivotal role in gene regulation as well as their networks. Throughout the last decade, miRNAs have been revealed to play important roles in tumor cell recruitment, progression, and metastasis (19). The miR 17-92 cluster expression, which cooperated with MYC to induce cancer growth in a B cell lymphoma mouse model, was the very first example (20). Certain miRNA also functions as tumor suppressors, for instance, the let 7 family suppresses tumor development and metastasis *via* targeting key oncogenic genes like high-mobility group AT-hook 2 (HMGA2), members of the RAS family (NRAS, KRAS, and HRAS), and MYC (21–23). As a result, cancer-related variations in the expression profiles of miRNA are emerging as promising diagnostic markers as well as the targets, for therapeutics, that are frequently linked to tumor growth and overall survival (19). Although particular miRNAs possess either an oncogenic or tumor-suppressive effect, multiple reports suggested a decreased miRNA expression universally in cancerous cells in contrast to healthy cells, implying that miRNA synthesis may be disrupted during tumorigenesis (24, 25).

**Figure 1:**
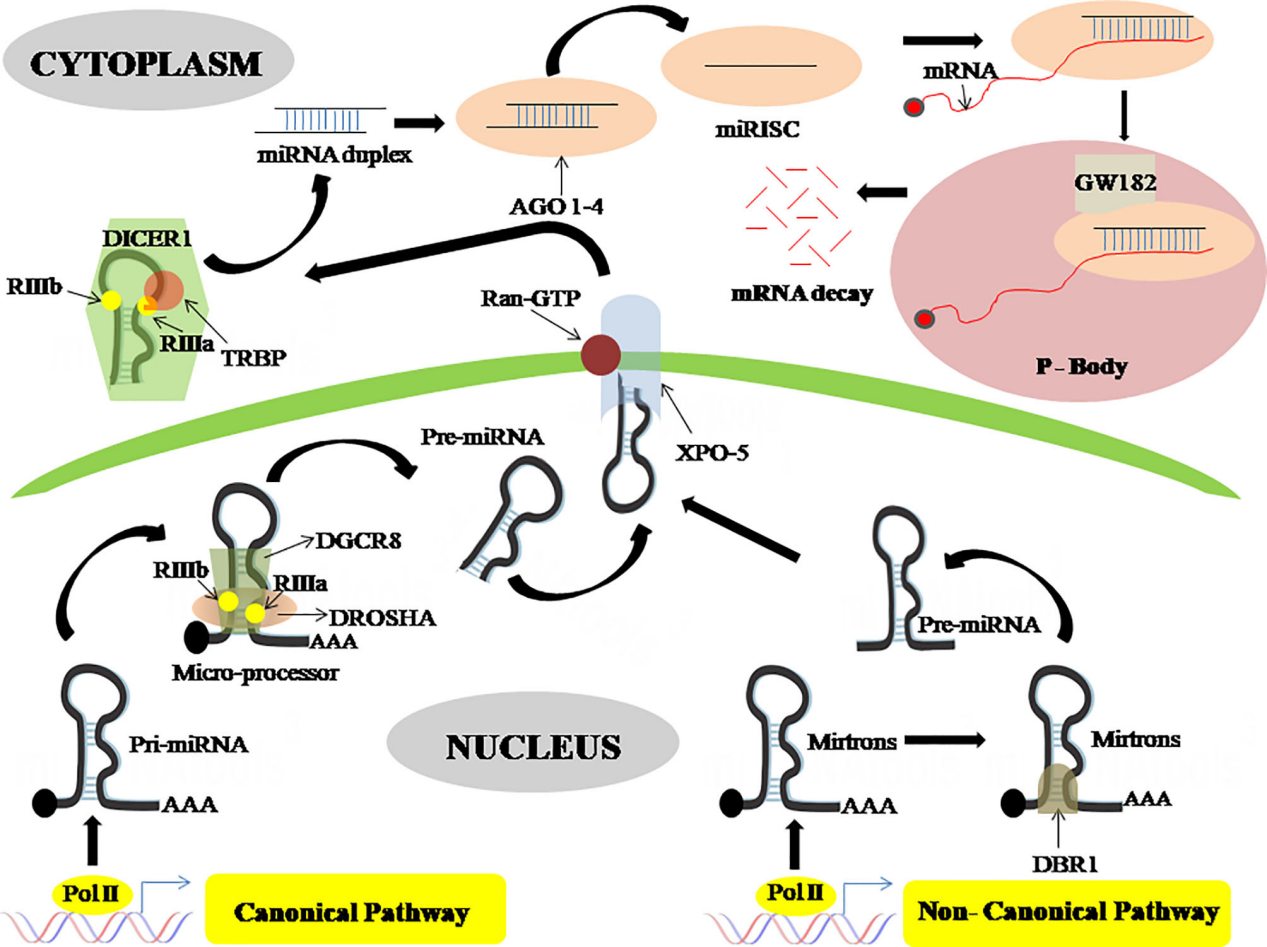
MicroRNA biogenesis through a canonical and non-canonical pathway. In the canonical pathway the pri-miRNA is processed by DROSHA and DGCR8 to form pre-miRNA which then is exported to the cytoplasm *via* exportin-5, wherein the cytoplasm this pre-miRNA is processed by DICER-1 which gives rise to miRNA duplex. This miRNA duplex is then incorporated into RISC together with an argonaute protein to form mature miRNA. This mature miRNA then binds with an mRNA in the processing body where mRNA decay happens. On the contrary, in the non-canonical pathway, the pre-miRNA is generated from mitrons by DBR1. This pre-miRNA is transported by exportin-5 and enters the canonical pathway. DROSHA, Class 2 ribonucleaseIIIenzyme; DGCR8, DiGeorge syndrome critical region 8 gene; DBR1, Debranching enzyme; XPO-5, Exportin-5; DICER, ribonucleaseIIIenzyme; AGO, Argonaute protein; RISC, RNA-Induced Silencing complex; P-Body, Processing Body.

Most of the miRNA genes are transcribed as pri-miRNA, made up of a hairpin loop structure which consists of a sequence of miRNA, by RNA polymerase II (Pol II) either as intronic clusters in the pre-mRNAs or as individual genetic elements, encoded within long non-coding RNAs (26). The biogenesis of miRNAs is carried out in two steps, first processed inside the nuclei and then in the cytoplasm (26, 27). DROSHA, an RNase type III enzyme, along with other related proteins comprises the microprocessor complex which catalyzes the nuclear event (26). This nuclear processing event leads to the synthesis of pre-miRNAs, which are ~70 nucleotides stem-loop-like precursor miRNAs that are then exported to the cytosol through the Exportin-5 (XPO-5) export receptor (28). The pre-miRNAs are later catalyzed in the cytosol by DICER, another RNase type III enzyme, which leads to miRNA duplex formation. These miRNA duplexes are then incorporated into RISC (RNA-Induced Silencing complex) along with another protein namely Argonaute (AGO), where only a single strand is chosen to form the mature miRNA (**Figure 1**) (29).

Also, there is a non-canonical miRNA biogenesis pathway that also produces functional miRNAs. Such as mirtrons which are produced through the pre-mRNA splicing process, while certain other miRNAs are produced from small nucleolar RNA (snoRNA) precursors, m^7^G pre-miRNA/Exportin1 pathway, t-RNA derived pathway, etc (16). Mirtrons are miRNAs, a byproduct of intron splicing, made by a non-canonical route that skips the Drosha cleavage step. Mirtrons go through lariat-debranching by DBR1, a debranching enzyme, then enter the conventional route at the exportin-5 level, therefore known as canonical mirtrons (**Figure 1**) (30).

Phosphorylation, ubiquitination, and sumoylation are some of the post-translational modifications of miRNA processing factors that can influence DGCR8, DROSHA, and/or DICER complex components. In another report, it was revealed that the regulation of miRNA biogenesis can also happen in a cell density-dependent manner (26).

## Role of MMPs in Cancer

MMPs are endopeptidases monitoring ECM’s physiological turnover and remodeling. While collagens, gelatins, proteoglycans, and elastin are among their substrates, they have a wide range of effects on many other proteins (31). Because MMPs digest a diverse array of substrates, their actions have a major impact on the extracellular environment, and if left uncontrolled, can lead to unnecessary ECM degradation (32, 33). MMPs consist of a predomain, catalytic domain, hemopexin domain and prodomain. MMPs are secreted as pro-enzymes, which are made inactive by interacting with a cysteine-sulphydryl group in the N-terminal (pro) domain with the zinc ion in the catalytic domain. The elimination of this association is known as the “cysteine switch,” and it is triggered by pro-hormone convertases (furin) (34). Another level of MMP regulation is performed by TIMPs that bind to the MMP catalytic site and regulate proteolytic activity. Nonspecific antagonists such as 2-macroglobulin, thrombospondin-1, and -2 can also inhibit MMPs (35). MMPs are divided into Collagenases, Gelatinases, Stromelysins, Matrilysin and membrane-type and non-classified MMPs subtypes. MMPs are crucial in the biochemical interplay between tumor and stroma. Stromal cells produce the majority of MMPs in the tumor microenvironment, bringing about ECM cleavage, thereby forming a path for cell movement from the tumor niche into adjoining areas and also releasing several bioactive compounds. Interaction of tumor cells with neighboring stromal cells is critical in facilitating cancer initiation and progression. Tumor cells secrete growth factors such as VEGF, EGF, FGF, interleukins, and IFN, which stimulate surrounding cells in the tumor tissues to release MMPs, allowing tumor cells to migrate (36, 37).

### MMPs in Cell Growth

Cancer cells are known for their uncontrolled proliferation. The tumor reaches this state in one of two ways: by being self-sufficient in growth-promoting signals or developing immunity to antigrowth signals. Cellular proliferation can be unchecked as a result of MMPs cleaving growth factor binding proteins, increasing their bioavailability, or activating growth factor receptors (38, 39). TGF-β It is activated by proteases like MMP-9, -2, -14, which leads to increased invasion and metastasis (40, 41). MMP-1 is found in stromal and epithelial cancer cells of invasive carcinomas and regulates cervical tumorigenesis and lymph node metastasis *via* the PPAR signaling pathway (42, 43). MMP-7 is implicated in cell proliferation, migration, and invasion, possibly through the wnt/catenin pathway (44, 45). Activation of the PKC pathway led to an increase in MMP-7 and 10 in cancer cells, indicating their involvement in cell proliferation and migration in OC (46). MMP-2 was shown to participate in OC cell proliferation *via* p38/MAPK pathway (47).

### MMPs in Apoptosis

Fas ligand binds to extracellular receptors like Fas receptors and activates intracellular caspases, resulting in the degradation of subcellular compartments, thus halting malignant spread. MMP activity inhibits apoptosis in malignant cells, by cleaving pro-apoptotic ligands or receptors (48).In human OC cells, downregulation of MMP-9 was shown to induce apoptosis and prevent proliferation (49). In another study, MMP-2 increased cell proliferation and reduced apoptosis in OVCAR3 (ovarian cancer cell line) cells, thereby lowering the effect of chemotherapeutic drugs on tumor cells (50).

### MMPs in Invasion and Metastasis

The tumor cells will subsequently enter the circulation and spread throughout the body by modulating MMP production (51). MMP-2 and -9 are the most prominent MMPs modulating cancer cell invasion. In both OC and CC, MMP-2 and -9 are implicated in cancer cell invasion and metastasis and are associated with poor survival (52, 53). Furthermore, MMP-2 promotes the attachment of metastatic OC cells to peritoneal surfaces by cleaving ECM and increasing their binding to integrin, as well as the OC cells’ propensity to metastasize (54). Similarly, in CC, an association of MMP-2 activation with αvβ3 integrin/MT1-MMP/TIMP-2 has been implicated in tumor cell migration (55). MMP-7 is the primary MMP linked with invasion and metastasis in EC (56). MMP-7 is also overexpressed in ovarian serous cancer tissues, where it increases cellular invasiveness by activating MMP-2 and -9 or by IGFBP breakdown, enhancing IGF concentration and cancer cell proliferation (57, 58).

### MMPs in Angiogenesis

The role of MMPs in angiogenesis is dependent on the neighboring environment, such as substrate abundance and MMP expression time points during angiogenesis (59). MMP-2 is a widely known influencer of vascularization during cancer development. In OC, MMP-2 expression was increased *via* PI3K/Akt and NFκB pathways, enhancing endothelial progenitor cell proliferation (60). Activation of PAR-1 *via* MMP-1 causes OC cells to secrete multiple angiogenic factors, resulting in cell proliferation, endothelial tube formation, and migration (61, 62). MMP-9 has a role in the release of VEGF from tumors (63). OC cells implanted into Mmp9^-/-^ nude mice showed significantly lower levels of VEGF in tumors, thereby contributing to angiogenesis (64).

## Regulation of MMPs by miRNAs

Considerable interest is seen in investigating post-transcriptional regulations of MMPs by miRNAs in recent times. Bioinformatics analyses have identified several miRNAs binding sites at the 3’UTR of MMP transcripts, thereby inducing mRNA instability or translational repression (11, 65). Studies have shown the participation of miRNA in regulating MMP gene expression thereby playing a key role in migration, differentiation, apoptosis, etc (66–68). These miRNAs either promote or repress malignant phenotype, acting as either oncogenic or tumor-suppressor, respectively. Oncogenic miRNAs (OncomiRs) are overexpressed in cancers whereas tumor-suppressor miRNA is downregulated, thereby leading to the onset of carcinogenesis, metastasis, and poor survival. However, there are conflicting pieces of evidence as if a miRNA behaves like an oncogene or tumor-suppressor in the tumor microenvironment. This review wishes to directly examine the effects of miRNAs towards MMP regulation in gynecological cancer development and disease progression (**Table 1**).

**Table 1:** Oncogenic and tumor suppressor miRNAs regulating MMPs during development of gynecological cancers.

Disease	microRNA	ExpressionLevel	MMPs Involved	Binding	Function(s)	References
Cervical Cancer	miR-1246	Upregulated	2/9	Indirect	Induces proliferation, tumor growth, cell migration, invasion, metastasis and EMT.	(69–72)
	miR-106a	Upregulated		Indirect		
	miR-183	Downregulated		Direct		
	miR200b	Downregulated		Indirect		
Cervical Cancer	miR-21	Upregulated	2/9	Indirect	Increased STAT3 decreased TIMP-3 and PTEN expression leading to cell invasion.	(73)
Cervical Cancer	miR-195-5p	Downregulated	14	Direct	Promotes proliferation and invasion by directly binding of miR-195-5p to 3’UTR of MMP-14 and modulating TNF-α pathway	(74)
Endometrial Cancer	miR-183	Upregulated	2/9	Direct	Promotes cell proliferation and invasion.	(75–77)
	miR-130b	Upregulated		Indirect		
Uterine Endometrial Stromal Carcinoma	miR-21	Upregulated	2	Indirect	Induces cell invasion and wound healing.	(78)
	miR-31	Downregulated		Indirect		
	miR-145	Upregulated		Indirect		
	miR-195	Upregulated		Indirect		
Ovarian Cancer	miR-92	Upregulated	2/9	Indirect	Promotes migration and angiogenesis by inhibiting VHL and upregulating HIF1α pathway genes.	(79, 80)
	miR-210	Upregulated		Indirect		
Ovarian Cancer	miR-205	Upregulated	2/10	Indirect	Promotes invasion *via* inhibiting TCF-21.	(81)
Endometrial Adenocarcinoma	miR-410	Downregulated	14	Direct	Promotes tumor formation.	(13, 82)
Endometrial Cancer	miR-195	Downregulated	2/9	Indirect	Promotes EMT by targeting GPER/PI3K/AKT.	(83)
Endometrial Endometroid Carcinoma	mir-22	Downregulated	2/9	Indirect	Induces cell proliferation and invasion.	(84)
Endometrial Cancer	miR-320a	Downregulated	3/9	Indirect	Inhibits TGFβ-induced EMT.	(85)
	miR-340-5p	Downregulated		Indirect		
Ovarian Cancer	miR-574-3p	Downregulated	9	Indirect	Promotes migration and invasion, inhibiting AKT, FAK and c-Src by targeting EGFR.	(86)
Ovarian Cancer	miR-29b	Downregulated	2	Direct	Induces cell migration by regulating crosstalk between OC cells and fibroblast.	(12, 87)
Ovarian Cancer	miR-1236-3p	Downregulated	2	Indirect	Promotes proliferation and invasion and EMT *via* VEGF.	(88)
Ovarian Cancer	miR-16	Downregulated	2/9	Indirect	Promotes migration and invasion *via* Wnt/β-catenin signaling pathway.	(89)
Ovarian Cancer	miR let-7d-5p	Downregulated	2/9	Indirect	Promotes proliferation by regulating p53 signaling pathway *via* HMGA1.	(90)
Ovarian Cancer	miR-1273g-3p	Downregulated	2/9	Indirect	Regulation of TNF-α and COL1A1.	(91)
Ovarian Cancer	miR-199a-5p	Downregulated	2/9	Indirect	Promotes cellular growth, proliferation and invasion *via* NF-κB pathway.	(92, 93)
	miR-9	Downregulated		Indirect		
Ovarian Cancer	miR-122	Downregulated	2/14	Indirect	Promotes EMT by targeting P4HA1.	(94)
Ovarian Cancer	miR-130b-3p	Downregulated	2/9	Indirect	Promotes EMT, cellular attachment and proliferation	(50, 95)
	miR-200	Downregulated		Indirect	through TGF-β signaling pathway.	
Ovarian Cancer	miR-17	Downregulated	2	Indirect	Promotes metastasis by regulating integrin α5 and β1.	(96)
Ovarian Cancer	miR-340	Downregulated	2/9	Indirect	Promotes metastasis and inhibits apoptosis *via* NF-x03BA;B1 activation.	(97)
Ovarian Cancer	miR-543	Downregulated	7	Direct	Promotes invasion by direct binding of miR-543 to 3’-UTR of MMP-7.	(98)

### OncomiRs

Several oncogenic miRNAs were found to be linked with gynecological cancer development and are involved in cell migration, angiogenesis, apoptosis, etc. (73, 78). Each miRNA has many different targets and modulate different signaling pathways in different cancer types (75, 99). The endogenous inhibitors of MMPs are known as tissue inhibitors of matrix metalloproteases (TIMPs). Disruption of MMPs/TIMPs balance occurs during multiple pathological conditions including cancer. In CC, miR-106a downregulates TIMP-2 through direct binding to its 3’-UTR region resulting in the induction of MMP-2 as well as MMP-9 expression and subsequently promoting cellular invasion, and migration (69). Alteration of TIMP-2 expression partly eradicates the invasion, migration, and MMP-2/9 expression in CC cells (34). Similarly, in HPV-induced CC, miR-21down-regulates TIMP-3, PTEN, and STAT3 expressions (61). Additionally, in uterine endometrial stromal sarcoma, miR-21 decreases the level of PTEN by directly binding to 3’UTR, leading to increased proliferation, invasion, decreased apoptosis, and metastatic potential thereby upregulating MMP-2 and -9 (73, 78). Epithelial-to-mesenchymal transition (EMT) is a crucial feature of cancer enabling cells to acquire mobility and translocate to distant sites. miR-183 promotes cellular proliferation and EMT in uterine EC by inhibiting CPEB1 expression and up-regulating MMP-9 expression. Studies revealed CPEB1 and MMP-9 as the direct target of miR-183, also a binding region for 3’UTR of MMP-9 is found at the seed region of miR-183 (75, 76).

Von Hippel Lindau (VHL), a tumor suppressor, targets HIF1α/2α by ubiquitination involving E3 ligase to proteasomal degradation. Loss of VHL results in the accumulation of HIF1α inside the nuclei and expression of HIF target genes which subsequently leads to oncogenesis (100). In OC, miR-92 inhibits VHL, which in turn de-repress HIF-1α. HIF-1α, in turn, stimulates VEGF by acting as a transcription factor together with p300 and p-STAT3 (99). Similarly, miR-210 is another important miRNA activated during the hypoxic condition and has a role in DNA damage response, mitochondrial metabolism, cellular proliferation, angiogenesis, and apoptotic cell death. Loss of VHL in OC stabilizes HIF-1α which in turn stimulates miR-210 expression inducing tumor aggressiveness (79).

DNA methylation/histone acetylation forms a complex framework for epigenetic regulation during cancer development. An altered methylation pattern is seen in cancer cells, both globally and CpG islands in the promoter region (101), leading to aberrant gene activity during tumorigenesis. In EC, different levels of miR-130b expression and its CpG methylation were linked to MMP-2/9 expression and EMT-related genes. Reversing miR-130b promoter hypermethylation decreased EC cell malignancy, suggesting that CpG island hypermethylation-mediated miRNA silencing contributes to carcinogenesis and is related to aggressive tumor behavior *via* increased MMP-2/9 expression, however, the mechanism behind the regulation of MMP expression by this miRNA is still unknown (77).

### Tumor Suppressor miRNAs

Tumor suppressor miRNAs are under-expressed during cancer progression and regulate cancer development by downregulating genes involved in tumorigenesis. The majority of ECs are accompanied by abnormal hormone signaling, where estrogen receptor α (ERα) behaves as oncogenic stimuli (102). Estrogen induction regulates cellular proliferation and subsequent invasion in EC and is accompanied by a downregulation of miR-22 in ER-α positive cell lines. Transfected miR-22 mimics into endometrial cells reduced the release of MMP-9 and MMP-2 thereby reversing 17β-estradiol (E2)-mediated progression of the cell cycle, cellular proliferation, and invasiveness of ERα-positive EC cells (84).

miR-200 family members have an enormous function in multiple cancer types (103–106). miR-200b plays a key role in regulating EMT and is correlated with cancer growth, proliferation, drug resistance in numerous diseases (107, 108). Cytoskeletal remodeling is the central event in the metastatic spread of cancerous cells. Actin structures facilitate cell migration and invasion, disruption of which leads to increased metastatic spread (109). In CC, miR-200b can suppress RhoE function, which regulates actin cytoskeleton and cell migration by altering cell motility by targeting MMP-9 thus suppressing EMT (70). Another report showed that downregulation of miR-200 family expression by TGFβ induced MMP-2, -9, and fibronectin 1 production and stimulated cancer cell attachment to human primary mesothelial cells (110). Catalpol induces miR-200 expression which sequentially inhibits MMP-2 expression levels, decreases cell proliferation, and accelerates apoptosis in OC cells (50). Similarly, TGFβ1 induced EMT was linked with decreased miR-320a and increased MMP-3 and -9 expressions in EC cells. Excessive expression of miR-320a or miR-340-5p substantially inhibited HEC-1A (endometrial adenocarcinoma cell line) cell invasion and migration through its binding to eIF4E mRNA 3’-UTR and diminished TGF-1-induced EMT properties (85). Another report suggested the involvement of miR-130b-3p in EMT, invasion, migration in cancer various types, mainly *via* the TGFβ pathway (111, 112). In OC overexpression of CMPK, cytidine nucleoside monophosphate kinase is seen, and CMPK knockdown dramatically decreases the cellular proliferation, invasion, and migration, along with MMP-9/-2 expression in epithelial OC. Downregulation of miR-130b-3p is seen in EOC which upregulates CMPK *via* the TGF-β signaling pathway (95).

Rak et al. showed a higher MMP-14 expression in endometrial adenocarcinoma tissue with a decrease in miR-410 level, suggesting a regulatory effect of miR-410 in modulating EC cell progression although the mechanism is largely unknown (13). Studies in odontoblast cells suggest the presence of a probable binding site for miR-410 on 3’UTR of MMP-14 (82). In lung cancer, miR-410 has a tumor-suppressive role by inducing apoptosis through downregulating JAK/STAT3/SOCS3 signaling pathway (113). Another miRNA, miR-195 has tumor-suppressive nature which negatively regulates cellular proliferation, migration, invasion, and promotes apoptosis (114–116). miR-195 overexpression ectopically decreased the viability, migration, and invasiveness of the endometrial carcinoma cell lines, along with the TIMP-2 upregulation and MMP-2/9 downregulation. miR-195 targets GPER (G protein-coupled estrogen receptor) and reduced the phosphorylation levels of PI3K/AKT, thus negatively regulating EMT in endometrial carcinoma (83). miR-195 also suppresses CC cellular proliferation, invasion, and migration through the TNF-pathway. The MMP-14 3’UTR binds to miR-195-5p directly through which its expression is directly inhibited. MMP-14 can modulate the expression of TNF-α. A downregulated miR-195-5p and an upregulated MMP-14 were noticed in CC (74).

miR-574-3p has an enormous role in cancer progression, EMT, metastasis, invasion, and chemosensitivity (117, 118). In epithelial OC, it inhibits the activation of AKT, FAK, c-Src, and MMP-9 by negatively regulating EGFR, inhibiting the cell invasion, and migration, and also increasing EOC cell sensitivity to paclitaxel and cisplatin (86). Different patterns of Let-7 family miRNAs were found in multiple cancers. In OC, let-7d-5p induces cell apoptosis and rescues chemosensitivity to cisplatin by targeting HMGA1 directly and thereby regulating the p53 pathway, MMP-2 and -9, and apoptotic pathway (90).

miR-17 is a highly conserved 6-membered gene cluster and is shown to have numerous roles in various pathways (119–121). In OC cells, it is seen to be downregulated thereby suppressing its inhibitory action of peritoneal metastasis *via* targeting integrin α5 and β1 and MMP-2 expression. miR-17 specifically binds to the α5 and β1 integrins 3’UTR region directly and decreases their expression. The addition of miR-17 to OC cells *in vitro* showed a significant decrease in adhesion and invasion (96). miR-29b is dysregulated in various cancers. It has a tumor-suppressing role in OC and is seen to be involved in tumor malignancy. It increases the α-SMA (mesenchymal cell markers) expression in fibroblasts which is a component of the cellular microenvironment that contributes to tumor malignancy by getting hyperactive and acquiring CAF (cancer-associated fibroblast) profile during carcinogenesis. These fibroblasts downregulate miR-29b expression in SKOV3 cells (ovarian cancer cell line), resulting in an increased invasion and migration. miR-29b can potentially target MMP-2 which is also found to be upregulated in OC (12). Studies in lung cancer metastasis also revealed the presence of a binding site of miR-29b at the MMP-2 3’UTR region through which it downregulates MMP-2 expression (87).miR-543 has been seen to be dysregulated in many cancers. It regulates proliferation, migration/invasion, EMT, metastasis, and many other pathways (122–124). miR-543 suppresses MMP-7 gene translation *via* the direct binding of MMP-7 3’-UTR whereas placental growth factor (PLGF), an angiogenic factor, represses the inhibitory action of miR-543 activating the MMP-7 mediated EMT and invasion in OC (98). Certain miRNAs have a dual role in carcinogenesis. Although previously stated that miR-183 is oncogenic, it is also seen to possess a tumor-suppressive function. In CC tissues, miR-183 expression was notably reduced whereas MMP-9 expression was elevated. The addition of miR-183 in-vitro resulted in a reduced invasion and migration of CC cell lines, *via* directly targeting MMP-9 and reduction of metastatic capability. A presence of a possible binding site of miR-183 was found at the 3’UTR regions of the MMP-9 gene (71).

## Predicting the Role of MMPs in Cancer Signaling Pathways

**Figure 2:**
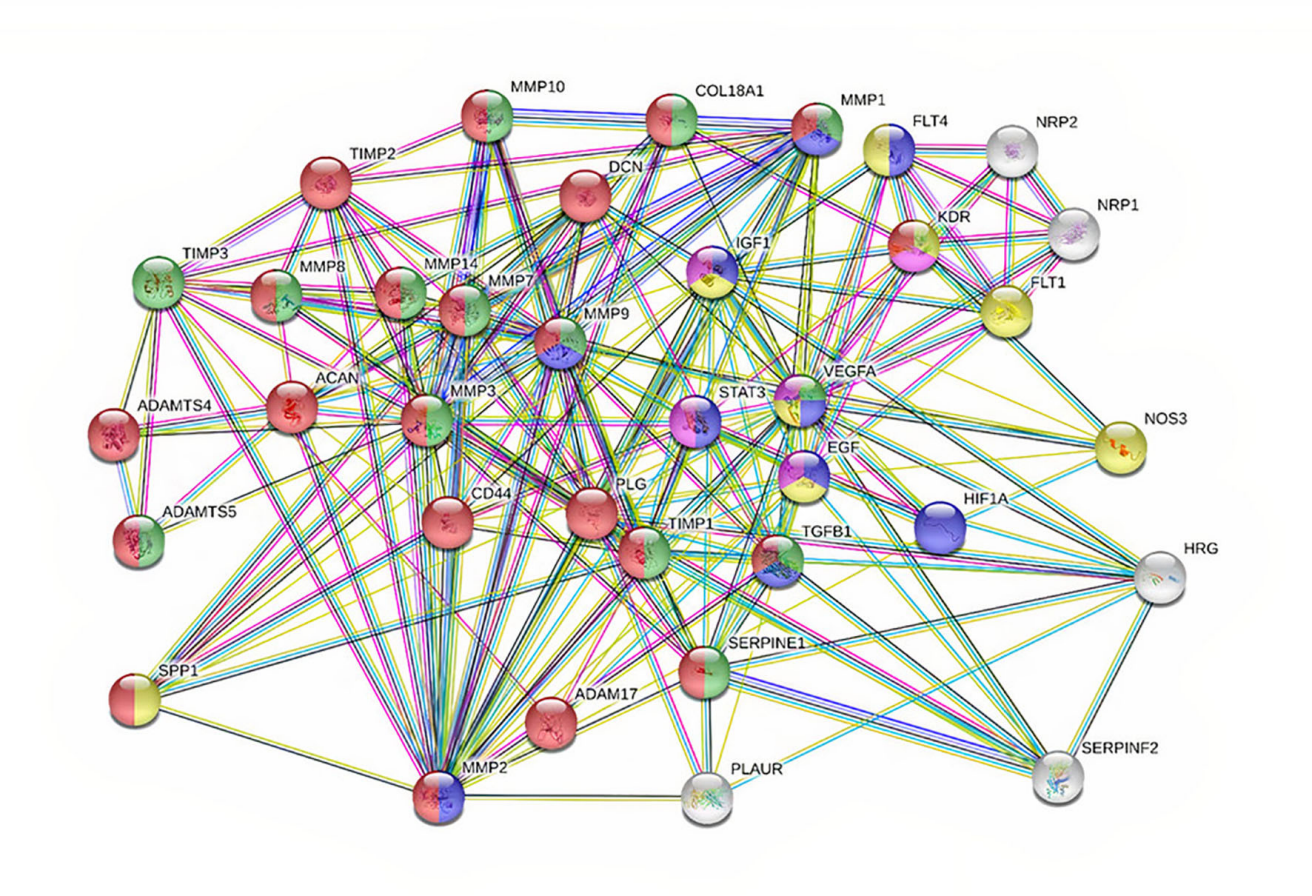
PPI network showing 36 associated proteins in cancer. The nodes are each candidate. Edges represent their interactions. The divisions with nodes are the shared functions. Blue symbolizes those that have a function in cell proliferation. Green and Red are the ones regulating ECM. Yellow are those with proliferation and angiogenesis.

To have a better understanding of the functions of MMPs and their regulation in cancer, an interaction plot has been created in String database (http://www.string-db.org) and analyzed in *Cytoscape ver 3.8.1.* Initially, miRwalk and miRmap database were used to find the miRNA and understand the regulating MMPs in gynecological cancer. The MMPs and their correlated genes were selected with k mean value of 0.23, neighborhood active interaction source, with a minimum confidence score of 0.45 and minimum stringency. As shown in **Figure 2**, MMP-9, -3, -7 and -2 are considered hub genes since most of the protein-protein interactions are seen among them. MMP-9, -7, -3, -2, -8 and -14 also show proximity to each other, hence they are correlated in each other’s biochemical activity. A significant positive correlation is also seen in MMPs interacting with genes viz; ADAM17, PLAUR, TGFB1, SERPINE1, STAT3, EGF and TIMP. Results showed a positive correlation with genes involved in tumorigenesis and extracellular matrix proteins (125–130). IGF1, VEGFA, STAT3, PLG, ACAN and TIMP-2 were found to directly regulate with MMP-3, MMP-9 and MMP-7, respectively (**Figure 2**).

**Figure 3:**
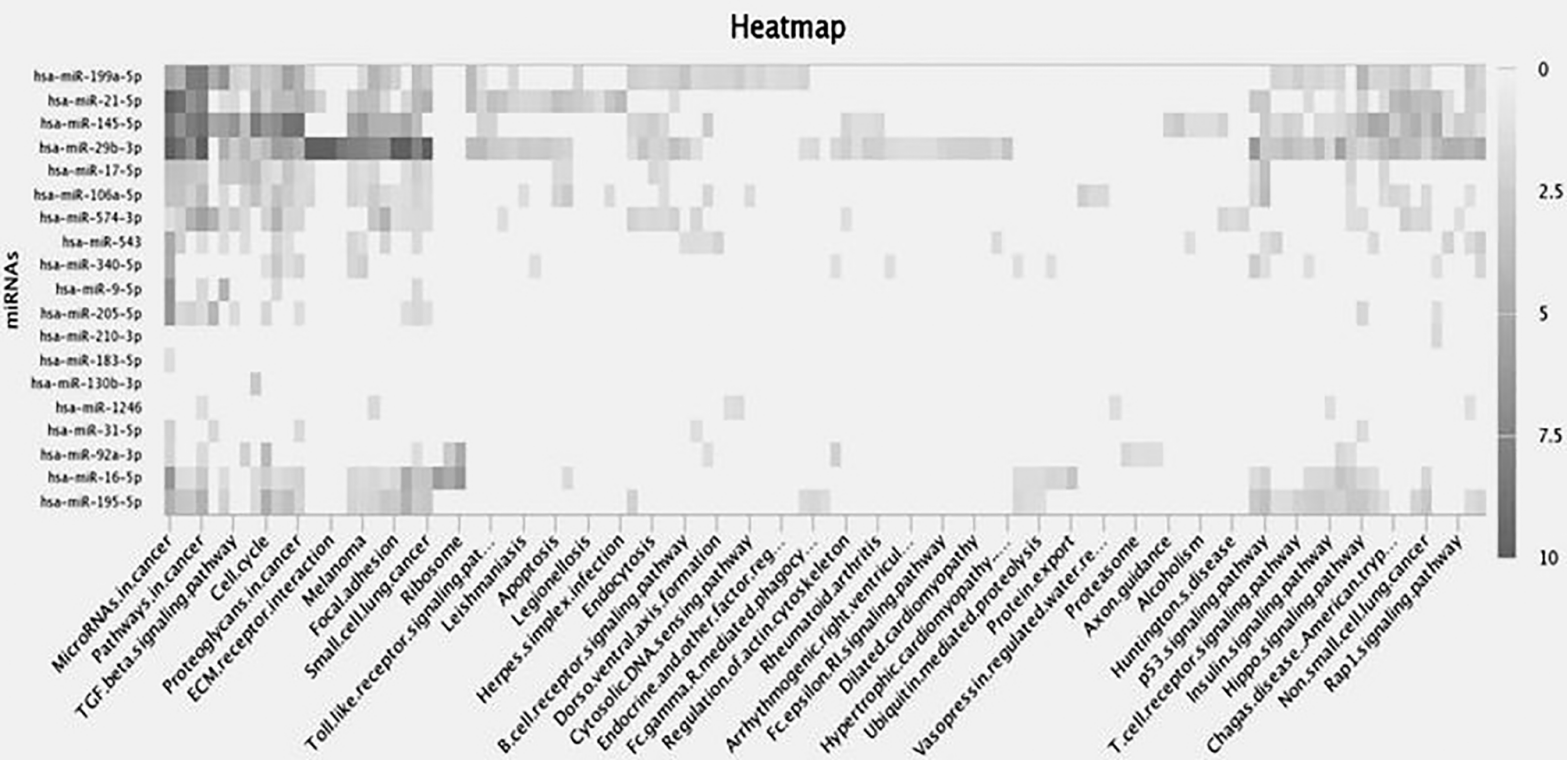
Heat map depicts differential expression of miRNAs in various biological processes. Rows represent enrichment results for the target miRNAs whereas columns show biochemical pathways. Each highlighted miRNA is correlated to the adjacent biological process in the black color gradient. The color of individual fields represents P-value of the enrichment results. The dark shade shows a strong correlation between miRNA and the target pathways, the light shades correspond to weaker ones, whereas transparent area explains no role of miRNA in that process.

Screening of the miRNA was performed from the miRNA library and enrichment analysis was performed to understand the cellular activity and biochemical pathways in the form of a heat map showing the association of miRNAs involved in signaling pathways was created in miRpath (https://tools4mirs.org/software/target_functional_analysis/mirtar/). Recent evidence suggests the participation of miRNA in regulating MMP gene expression and is associated with key physiological pathways like TGF β, Rap1, Toll-like, Hippo, B cell and T cell receptor signaling pathway (131–136) (**Figure 3**). miRNAs regulate the actin cytoskeleton, which works synergistically on MMP regulation during cancer growth and metastasis (137, 138). As seen from th heatmap, among the miRNAs reported to regulate MMPs in gynecological cancer, miR-199-5p, miR-21-5p, miR-145-5p and miR-29b-3p have shown the highest correlation with cancer-related signaling pathways (**Figure 3**). miR-145-5p and miR-21-5p are associated with *TGF βand Hippo signaling pathway* whereas miR-29b-3p regulates *FAK pathway, Insulin pathway* and *p53 signaling pathway*, along with ECM receptor interactions and is also shown to play a crucial role in small cell lung cancer and melanoma (**Figure 3**). From literature studies, we found that miR-29b directly binds to MMP -2 3’UTR and regulates their expression in OC (12). Prudent manipulation of these miRNAs can therefore regulate MMP production in cancer cells and can act as antitumor agents.

## miRNA-Based Anti-Cancer Therapeutic Strategies

miRNA-based therapeutic protocols for regulating gene expression can be divided into two main strategies: miRNA anti-sense therapy and miRNA replacement therapy. Inhibition of oncomiRs synthesis can be achieved by using miRNA inhibitors or oligomers and, on the other hand, enhancement of miRNA activity can be achieved by replacement of oncomiRs with the viral vector-mediated introduction of tumor suppressor miRNAs in a cell-specific manner for reprogramming target cells. Strategies to inhibit oncomiRs biogenesis by small-molecule inhibitors, antagomiRs, miRNA sponges, miRNA masking and approaches for replacement of miRNAs, including lentiviral vectors, tumor-suppressor miRNA mimics, CRISPR/Cas-like genome editing tools, are currently being investigated as potential cancer therapeutics.

Locked nucleic acid (LNA), a class of high‐affinity bicyclic RNA analogs, can detect miRNA in tissues and inhibit their function *in vitro* and *in vivo* studies. *Miravirsen*, a short locked nucleic acid complementary to miR-122 (Roche/Santaris) is the world’s first miRNA drug candidate in phase II clinical trials for hepatitis C virus treatment, along with RG-101, an N-acetylgalactosamine-conjugated anti-miR targeting miR-122 (139, 140). Furthermore, tumor suppressor miRNA replacement has been explored utilizing miRNA mimics/lentiviral vectors producing miRNA, which may influence endogenous miRNA expression (141–144). As an alternative to lentiviral vectors that show off-target effects, nonviral miRNA delivery techniques like polyethyleneimine (PEI)-based nanoparticles, liposomes, polymeric micelles, and dendrimers have been proposed. MRX34 was the first miRNA replacement therapy in modified liposomes to enter clinical trials, restoring a tumor suppressor miRNA, miR-34, with promising outcomes in stage I trials (139).

Several potential small molecule drugs targeting enzymes involved in miRNA biogenesis have been identified using comprehensive compound library screening. miR-21 is upregulated in most cancers and suppression of PTEN by miR-21 can contribute to chemoresistance *via* activating the Akt/ERK pathways (145). Screening for small molecules modulating miR-21 activity resulted in the discovery of a novel etheramide backbone which led to a reduction in CC cell proliferation and tumor growth, as well as the activation of apoptosis by activating caspase-3/7 (145).

miRNA sponges are artificial transcripts containing several complementary binding sites for one or more miRNA of interest and can block the activity of multiple miRNAs sharing the same seed sequence. miR-9 reduced the expression of KLF17, CDH1, and LASS2 (tumor suppressor genes). A DNA sponge with four miR-9 binding sites was demonstrated to effectively inhibit miR-9 activity, restoring natural expression of KLF17, CDH1, and LASS2 (146). Researchers are also focusing on utilizing CRISPR/Cas9 gene-editing system for miRNAs inhibition. In human colon cancer cell lines targeting of miR‐17/miR‐200c/miR‐141 loci was done using CRISPR/Cas9 resulting in decreased levels of mature miRNA and low off‐target effects (147).

Combination strategies based on the co-administration of miRNA targeting agents along with antitumor drugs have been observed to eradicate drug-resistant tumor cells to treatment and have greater anticancer effects. Nano-liposome-based delivery of miR-205 mimic was shown to sensitize the tumor to radiation therapy in breast cancer xenograft model (148). In another example, PDL1 expression in tumor cells was decreased when mir-34a mimics (MRX34) were combined with radiation (149). Therefore, combining miRNA replacement therapies with conventional anticancer drugs reveal excellent results and presents a novel possibility of chemotherapeutic treatment regimens.

The capacity to target several genes in a particular pathway and efficiently build novel therapeutic components are two advantages of miRNA-based treatment. Given that a single miRNA may regulate multiple MMPs and their downstream signaling pathways amplifies the scope of utilizing miRNAs to act as an attractive candidate for anticancer treatments. However, this also invites additional problems of non-specific target inhibition by miRNAs. Targeting MMPs has been clinically challenging due to the non-specificity and musculoskeletal toxicity of the inhibitors (150). Therefore, precision medicine designed to target the MMPs increased in a particular tumor in a patient might show a potential resolution for this issue.

Even though there are no FDA-approved miRNA therapy candidates for medical intervention to date, potential candidate drugs are in clinical development or are in phase I and II clinical studies (151). Nanoparticle-based, tissue-specific miRNA-drug delivery to a particular lesion in a patient, can improve solubility and efficacy of the medicine while avoiding contact with healthy tissues. Intratumoral injections of miRNA-based therapeutics directly into the pathogenic site can improve bioavailability, target specificity, effectiveness, and reduce adverse effects in cancer-related diseases (152, 153). Computational deep-learning-based approaches for accurately predicting human miRNA targets at the site level in patients have enabled the use of huge multi-omics data and increased the robustness of prediction models. It is critical to design a good delivery mechanism with high specificity for targeting cells to execute miRNA replacement therapy. As a result, miRNA replacement therapy may be a unique and appealing treatment option for a variety of cancers, and it is vital to research how to carry the appropriate miRNA based on the kind of cancer.

## Conclusion and Future Prospects

**Figure 4:**
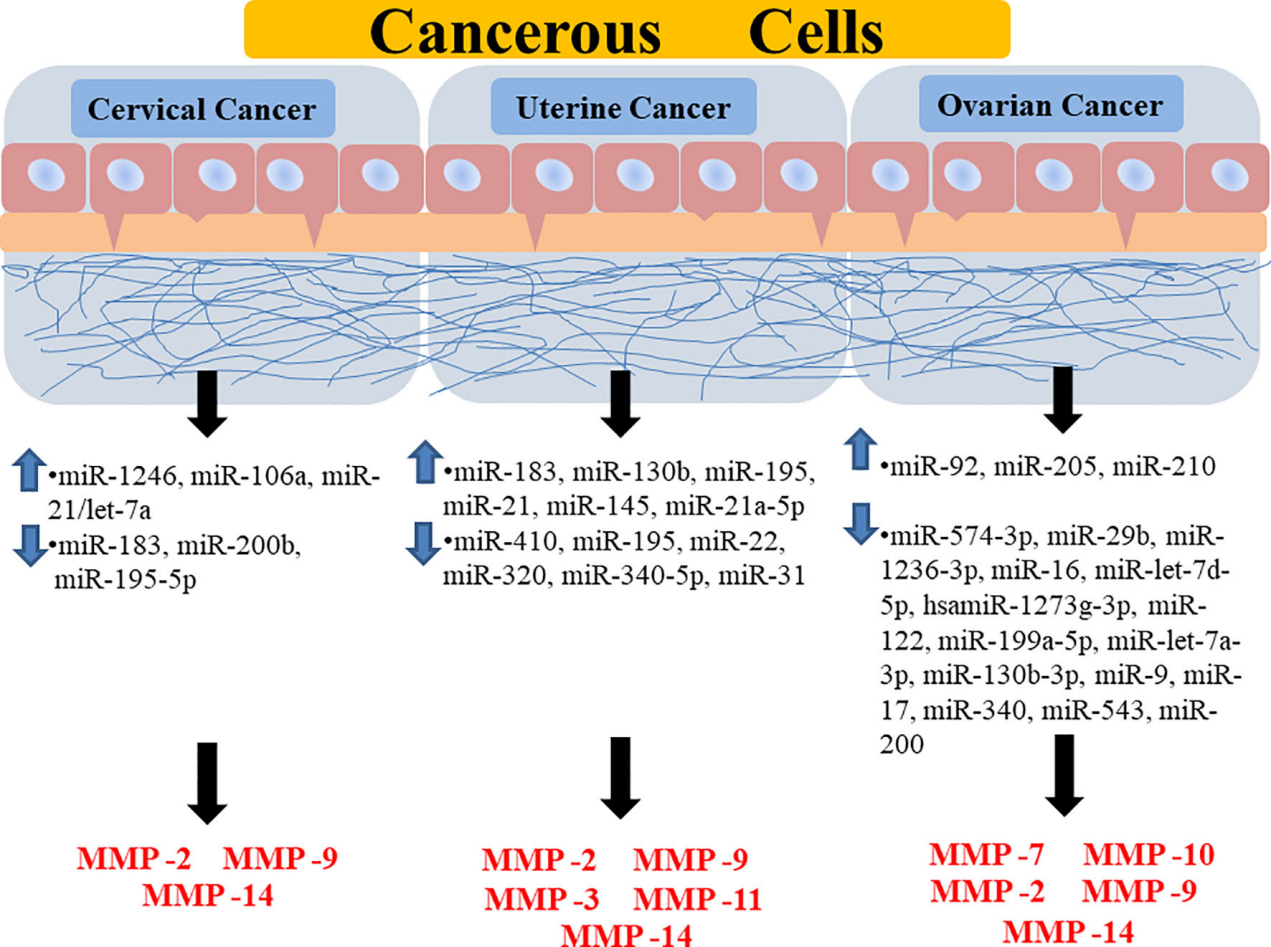
Diagrammatic representation showing the regulation of different MMPs through different miRNAs in various forms of gynecological cancer. It is showing how the upregulation or downregulation of certain miRNAs is promoting the expression of certain MMPs in a specific type of gynecological cancer. Highlighted miRNAs are highly correlated to major signaling pathways and target MMP-9/2 activities. MMP, Matrix Metalloprotease; miR, MicroRNA.

MMPs are powerful regulators of cellular proliferation, differentiation, angiogenesis, migration, and apoptosis. MMPs are appealing targets for the creation of selective inhibitors with high therapeutic potential. However, all of the clinical trials in advanced cancer patients with MMP inhibitors were unsuccessful. Numerous MMP inhibitors, including small molecules and blocking antibodies, have been produced as drug candidates to attenuate MMP production but most of their effects tend to be majorly nonspecific. Since MMPs contain similar active sites and play multiple crucial roles in important biological processes, making it is challenging to construct highly selective MMP inhibitors with low toxicities. Therefore, to increase the clinical utility of MMPs for tumor therapy, new MMP inhibitors should be able to individually regulate individual MMPs as well as manage a network of interlinked molecules. The ability of miRNAs to regulate potentially hundreds of genes in a cell-specific manner makes it a powerful target for anticancer treatment (**Figure 4**). Since miRNAs may target MMPs more selectively without interfering with the structural similarities of MMP catalytic domains, miRNA-mediated MMP regulation may lead to the creation of MMP inhibitors. Furthermore, miRNAs may target several molecules, often in the context of a network, making them particularly effective at controlling various biological processes essential to malignant tumors. Comprehensive inter-atomic analyses of miRNAs involved in regulating signaling pathways associated with cancer development and progression might aid in establishing druggable targets for antitumor treatment. Therefore, targeting such miRNA will not only help in understanding their functions but also the underlying cause of several gynecological disorders arising today. For probing miRNA-MMP as an anticancer treatment, proper validation and optimization of miRNA functional role are required in the clinical system, xenograft and orthotopic models to elucidate a detailed understanding of their efficacy in carcinogenesis and for their journey from bench to clinic. Pharmaceutical companies are constantly developing new miRNA-MMP therapies of low cytotoxicity and limited side effects. Whether new technologies targeting miRNAs that regulate MMPs can successfully be employed to delay or stop cancer progression remains to be seen.

## Author Contributions

Authors AP and YB were responsible for constructing the title, performing literature study, writing, illustration, and table preparation. SS and AS had taken the initiative of the work and gave their feedback on the study. PS carried out in silico studies and critically reviewed the article. All authors contributed to manuscript revision, read, and approved the submitted version.

## Funding

AP and YB are recipients of CSIR and UGC Fellowship, Government of India, respectively. PS gratefully acknowledges the financial support of DBT-RA Program in Biotechnology and Life Sciences, Government of India.

## Conflict of Interest

The authors declare that the research was conducted in the absence of any commercial or financial relationships that could be construed as a potential conflict of interest.

## Publisher’s Note

All claims expressed in this article are solely those of the authors and do not necessarily represent those of their affiliated organizations, or those of the publisher, the editors and the reviewers. Any product that may be evaluated in this article, or claim that may be made by its manufacturer, is not guaranteed or endorsed by the publisher.

## Abbreviations

miRNAs, microRNAs; MMP, matrix metalloprotease; ECM, extracellular matrix; CC, cervical cancer; OC, ovarian cancer; EOC, epithelial ovarian cancer; EMT, epithelial-mesenchymal transition; AGO, Argonaute.
